# The use of some nanoemulsions based on aqueous propolis and lycopene extract in the skin's protective mechanisms against UVA radiation

**DOI:** 10.1186/1477-3155-9-3

**Published:** 2011-02-04

**Authors:** Monica V Butnariu, Camelia V Giuchici

**Affiliations:** 1 Exact Sciences Department, Banat's University of Agricultural Sciences and Veterinary Medicine from Timisoara, Calea Aradului no.119, 300645 Timisoara, Romania; 2 Inspectorate for quality of seed and planting materials, Delamarina Victor Vlad no. 3, 300077 Timisoara, Romania

## Abstract

**Background:**

The use of natural products based on aqueous extract of propolis and lycopene in the skin's protective mechanisms against UVA radiation was evaluated by means of experimental acute inflammation on rat paw edema. The aim of the present study was to evaluate the harmlessness of propolis - lycopene system through evaluation of skin level changes and anti-inflammatory action. The regenerative and protective effect of the aqueous propolis and lycopene extract is based on its richness in biologically active substances such as: tocopherols, flavonoids, amino acids, polyunsaturated fatty acids, the chlorophyll pigment, all substances with strong antioxidant activity, that modify the oxidative stress, mainly by reducing the prooxidant processes and enhancing the antioxidant ones. These substances participate in the synthesis of prostaglandins and phospholipids components of cell membrane thus enhancing skin protection mechanisms.

**Results:**

The experimental systems offered a sustained release of the drug, *in vitro*, for aim eight hours. The prepared formulations aim did not reveal a deteriorating effect on tissues. They proved a better therapeutic efficiency Compared to standard suspension, they provided a better therapeutic efficiency coupled with extended time interval of tested parameters (24 hours). Preliminary examination of tissues showed that the experimental formulations did not irritate. Local application of propolis and lycopene aqueous extract nanoemulsion has a high potential both regarding its efficiency (the analgesic effect) and therapeutic safety.

**Conclusions:**

This study demonstrates that propolis and lycopene extract nanoemulsions, preparations contains active substances, can confer better therapeutic effects than those of the conventional formulations, based on local control-release of dozed form, for a longer period of time, which probably improve its efficiency and skin acceptance, meaning a better compliance. The information obtained in the present study suggests that administration of propolis and lycopene aqueous extract nanoemulsion is safe. The preparation can be useful for further preclinical studies lycopene embedded in aqueous propolis extract to be used in pharmaceuticals (targeted medical therapy).

## Background

In recent years, it has been noticed that the incidence of skin cancer has increased alarmingly. Exposure to UV irradiation has instantaneous effects (erythema and pigmentation) and delayed effects (premature skin ageing and different forms of cancer) [1]. UVB radiation has a stronger energy compared to UVA radiation and is absorbed directly by a series of cellular constituents, such as nucleic acids, proteins and urocanic acid. UVB radiation has also mutational effect [2]. UVA radiation penetrates easily through epidermis and acts on its basal proliferative layer and even on blood components of the dermis [3,4]. It acts indirectly on the cellular constituents, through oxidative mechanisms that forma reactive oxygen species [5,6]. Reactive oxygen species have a relative short lifespan, nevertheless are highly reactive with the vast majority of cellular components: nucleic acids, proteins, lipids, polysaccharides. Frequently their action induces irreversible modifications [7,8]. UVA radiation acts upon biological environments through oxidative mechanisms, correlated with the formation of reactive oxygen species: singlet oxygen, hydroxyl radicals, superoxide anions, hydrogen peroxide [9]. Nucleic acids and proteins adsorb poorly radiation however but the initial event triggering biological effects is made up of absorption of UVA photons by different chromophores in the cellular environment such as: quinones, steroids, porphyrins, proteins with flavin coenzymes and heme group (cytochrome, peroxidase, catalase) [10]. Many cellular components are targed by reactive oxygen species generated by UVA irradiation [11,12].

Hydroxyl radicals react with almost all cell molecules types: carbohydrates, phospholipids, nucleotides, organic acids and amino acids. On enzymes, the effect of reactive oxygen species results in catalytic capacity reduction, often determined by sulphhydryl oxidation and modification of amino groups by malonylation [13]. Organisms are protected against reactive oxygen species attack in several ways: cellular compartmentalization, protection afforded by antioxidant compounds and enzyme systems, their ability to develop adaptive responses inducible under oxidative stress conditions. Repair and *turnover *processes help to minimize these [14,15]. Under normal circumstances there is a balance between antioxidant systems and reactive oxygen generative systems. Lack of balance in favor of prooxidant systems causes the apparition of oxidative stress, with pathological implications [16]. Skin is the organ most exposed to solar radiation [17]. Skin presents a series of structures with a protective role, such as stratum corneum and melanin. Superficial corneum layer functions as optical barrier by reflection, scattering and absorption of incident radiation. Larger part of UVA radiation penetrates deeply into the skin, to dermis [18]. UVA radiation can be absorbed by different components of the blood, at the level of blood vessels. UVA radiation acts as inducer of enzymes responsible for polyamine synthesis. An additional mechanism of epidermis protection is to stimulate skin pigmentation with melanin [19]. The protection mechanisms are established and inducible protections at the skin level. Inducible defence mechanisms were not identified at epidermis [20], but was identified in dermis, where increased heme oxygenase, which are correlated with an increase in ferritin levels [21]. Pharmaceutical and cosmetics industries have launched a wide range of substances that act as filters capable of absorbing UV photons [22]. Photoprotection products are characterized by the protection factor. An accurate assessment of the effectiveness of photoprotection products should be based on their ability to inhibit the isomerisation reaction of urocanic acid and prevent accumulation of the protein [23]. "Quantum dot" nanostructures have been used (nanoparticles with quantum properties and ability to change size according to light emission). Another reason for the use of these products is their ability to "connect" many substances, thanks to a large surface area, and easy transport due to their small sizes (10 to 100 nanometers). These substances can also remain and accumulate preferentially at skin level, facilitated by surface drainage [24]. Currently, nanomedicine is seen not only as a possible and promising path to an early and effective treatment, but also a possible way to prevent certain types of diseases [25].

## Results

### Characterization of lycopene extract

**Figure 1:**
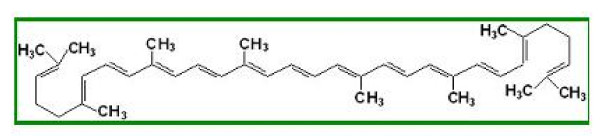
**Molecule of lycopene (chemical structure)**.

**Figure 2:**
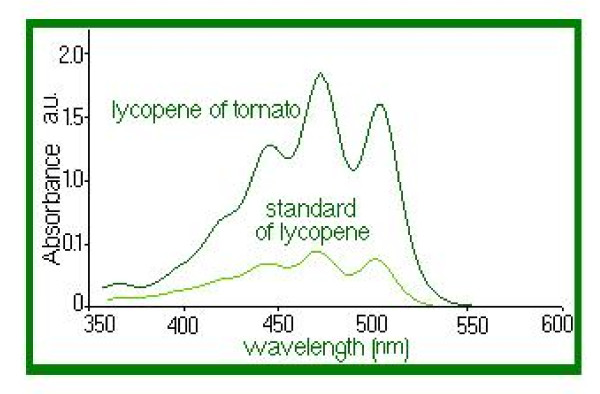
**UV-VIS spectrum of lycopene extracted from tomatoes and of standard of lycopene**.

**Figure 3:**
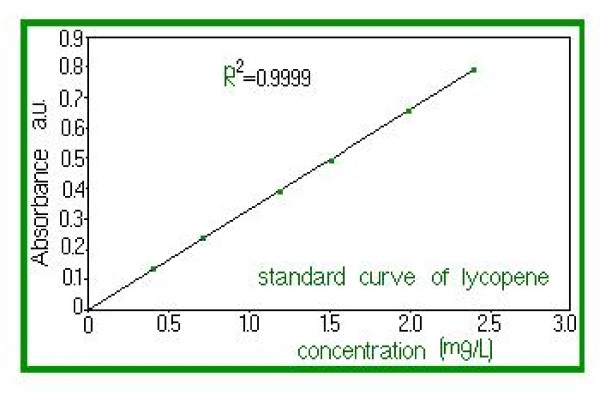
**The calibration curve of lycopene standard**.

Lycopene (Figure 1) belongs to the class of natural pigments, called carotenoids, with a role in protecting the body from the destructive effects of oxidants. Lycopene neutralizes the negative effects of free radicals. Furthermore, recent studies have shown that lycopene has a significant potential to counter free radicals in comparison with β-Carotene. Validation was confirmed by application of standard techniques lycopene determination. Aqueous extract of propolis has a high concentration of polyphenols and is standardized in polyphenol carboxylic acids (caffeic acid), responsible, among other active substances, for its healing and anti-inflammatory action upon tegument affected by dandruff and seborrheic dermatitis. Thus, the antimicrobial and anti-inflammatory action of lycopene is enhanced and regeneration of skin affected by fungal and/or microbial infections is stimulated. The product formulation also considered physiological aspects of the skin, resulting in a product with low allergenic potential and high degreasing capacity. Because of the nanoemulsion pharmaceutical form, it has several advantages over other topical products with similar action. Thus, in contact with skin it quickly releases active substances due to its good adherence to skin and close contact it has a high therapeutic efficiency, easy administration low allergenic potential, good local tolerance and an increased viscosity, allowing the required concentration in bioactive compounds. Figure 2 clearly shows the absorption spectrum of the extracted lycopene solution. The absorption spectrum very closely coincided with the three peaks characteristic of trans-lycopene (λ = 446, 472, 505 nm). Any analytical method (bio analytical, in particular), in order to be validated, must demonstrate first that it is specified in relation to existing endogenous substances in the biological matrix, to metabolism products and reagents used in the sample preparation. For that, the specificity of this method was verified using six different sources of blank solutions. We aimed to see if there was any endogenous interference at the retention times of the experimental analytes. The linearity of the method was verified by the method of smallest squares, on the 0-3.0 mg/L lycopene domain, using internal standard calibration as calibration model. Lycopene area and standard area ratio were calculated for seven levels of concentration in the selected domain and used for calibration curves. For each calibration point we examined the distribution, the relative percentage deviation of recalculated concentration from calibration curve equation. The calibration model was considered correct if residuals were within boundaries of ± 20% at lower limit and ± 15% at other concentrations and did not have a trend of increase or decrease along with concentration. Correlation was considered linear at a value of the determination coefficient greater than 0.99, as seen in Figure 3. Accuracy, expressed as relative percentage deviation of measured concentration in relation to the a obtained concentration, and precision, expressed as standard relative percentage deviation or coefficient of variation CV%, were determined at three concentration levels. Both, precision and accuracy were determined on the same day, based on five measurements on five different samples at each concentration, and accuracy and precision on different days based on the analysis on different days of five standard samples at each of the three levels of tested concentration. The lowest limit of quantification was considered the lowest concentration on the calibration line with an accuracy and precision within ± 20%. Retrieval was assessed at four concentrations, including the lower limit of quantification, comparing the response obtained after application of UV radiation with that obtained with a standard solution of the same concentration in water and similarly processed as biological samples.

### FTIR analysis

**Figure 4:**
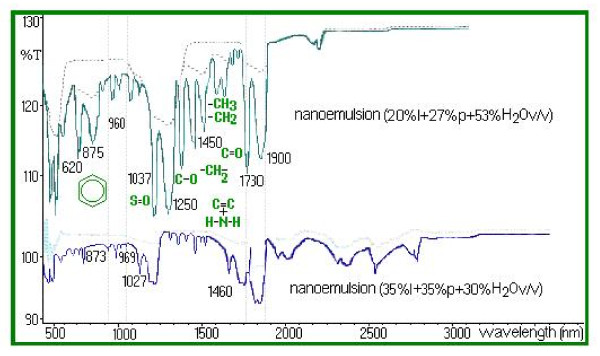
**IR spectrum of nanoemulsions in KBr disc, obtained from experimental analysis**.

All FTIR analysis is considered technically "non-destructive" therefore further analysis can be performed. Composition analysis by FTIR spectroscopy (Fourier Transform Infrared) allows quantitative estimates and the study of links nature that appear during the nanoemulsions process. Following FTIR spectra analysis, no significant differences were apparent between the products. As shown in Figure 4, differences were found in FTIR spectra in the region 900-500 nm regarding experimental conditions and the resulting products. IR spectra highlight the presence of -CH_2_- groups illustrated by the characteristic bands at 1450 cm^-1 ^and 1460 cm^-1 ^respectively. The nanoemulsion ester group causes the appearance of characteristic frequency bands at 1700 cm^-1 ^(-C = O stretching) given by a larger amount of propolis and lycopene. Increase in propolis content (the 30% option) determines the appearance of new frequency bands characteristic of the carbonyl group (1900-1600 cm^-1 ^-C = O stretching). Growth leads (in propolis) to the disappearance of intense bands at 750-1280 cm^-1^, assigned to ring vibrations: 1235-1280 cm^-1^, 810-905 cm^-1 ^and 805-875 cm^-1 ^and can be attributed to characteristic methyl bands shielding or to the dissolution of certain groups during the process of obtaining the nanoemulsion. In the case of the bands group in the range of 1150-1250 cm^-1^, characteristic of ester groups (-C-O stretching), an increase in the intensity of the bands is observed, which is reflected in the decrease of its transmittance from 96% to 56%. This is also explained by the increasing of the quantity of propolis from 27% v/v to 35% v/v in the nanoemulsion. High lycopene content of 35% has the effect of increased intensity of certain bands at 1450 cm^-1 ^(-CH_3 _and CH_2 _= strain) reflected in the decrease of the transmittance from 92% to 70.846%. An intensity increase was observed at 1730 cm^-1 ^(-C = O stretching) a characteristic of unsaturated esters. This growth is highlighted by the decrease of transmittance from 97.95% to 47.126% and is explained by 35% propolis content. The intensity significant increase of nanoemulsion bands characteristic is attributed to the increase of distance interactions between atoms and molecules, through altered angles between the links. In the case of experimental formulated nanoemulsions, for second formulation spectral bands intensity was reduced, which is consistent with the reported kinetic data (Table 1). Table 1 shows correlation coefficients of data regarding the release of formulated nanoemulsions, obtained from the Higuchi model, zero order kinetic and release exponent values (n) obtained from the equation Mt/Mo = ktn regarding the prepared nanoemulsions based on propolis lycopene (n = 3). During these experiments no changes were observed in the organoleptic properties of experimental formulations and pH value of the two nanoemulsions remained within the limits of ± 0.3 pH units. No significant changes were noticed in terms of particle size (p < 0.05).

**Table 1:** Data correlation coefficients regarding the release of active constituents of propolis lycopene systems

Sample	"n"	R	Higuchi Model		Zero-order kinetics	
			
			k (% h^-1/2^)	R	k (% h^-1^)	R
formulation with 20% lycopene, 27% propolis, 53% water vol./vol (nanoemulsion 1)						

Newly prepared			4.45	0.997	0.62	0.994
			
After one week	0.80	0.971	3.18	0.998	0.70	0.998
			
After two weeks			4.21	0.982	0.78	0.998
			
After three weeks			4.16	0.987	0.77	0.999

formulation with 35% lycopene, 35% propolis, 30% water vol./vol (nanoemulsion 2)						

Newly prepared			3.47	0.983	0.71	0.998
			
After one week	0.98	0.980	3.87	0.996	0.67	0.995
			
After two weeks			3.68	0.999	0.62	0.999
			
After three weeks			3.48	0.989	0.68	0.990

### The properties of the two experimental formulations

Micrometric properties of the two experimental formulations are shown in Table 2. This experiment highlighted the improved performances of the se emulsions without side effects and adverse reactions by replacing synthetic chemicals with natural products. It has been documented, that some UV radiation absorbers can be partially degraded therefore causing skin alterations while exposed to UV influencing the effectiveness of sunscreen protection. For information on UV absorbers, the nanoemulsions SPF *in vitro *parameters were investigated. UVA radiation, the UVA/UVB ratio and *in vitro *SPF were measured using the Diffey and Robson method. The efficiency of these products is measured by a coefficient, index or protective factor, noted SPF (sun protection factor) or PI (protection index), which is the ratio of the minimum dose of solar radiation that causes lowest skin redness, after and before skin application of these preparations.

**Table 2:** Micrometric properties of the two formulations tested

No.	Micrometric property	Nanoemulsion 1	Nanoemulsion 2
1	Response angle	35.60 ± 1.33	51.94 ± 1.68

2	Density of the nanoemulsion (merged)	0.38 ± 0.03	0.41 ± 0.02

3	Density of the nanoemulsion	0.53 ± 0.07	0.42 ± 0.02

4	% Porosity	42 ± 1.3	68 ± 2.4

Each value represents the average (± SD) of three independent determinations.			

### The higher the SPF values, the more efficient the photoprotection

**Figure 5:**
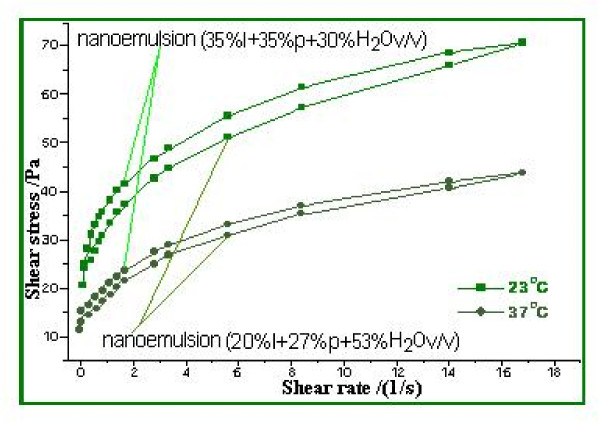
**Dependence between the absorbance parameters of the two formulations**.

**Figure 6:**
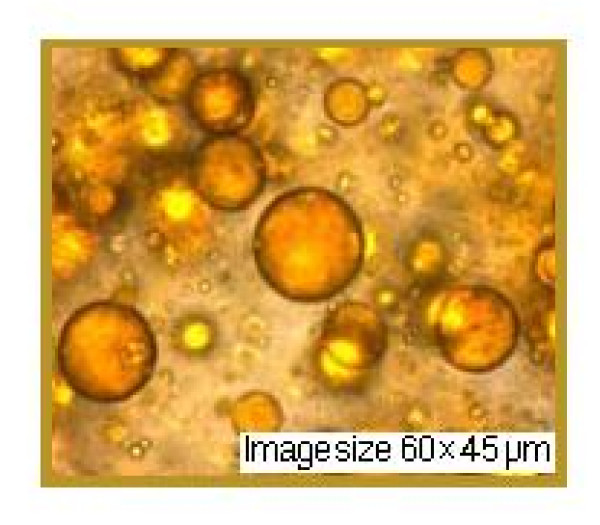
**Nanoemulsion 2 layer (aqueous extract of propolis and lycopene) after one week**.

**Figure 7:**
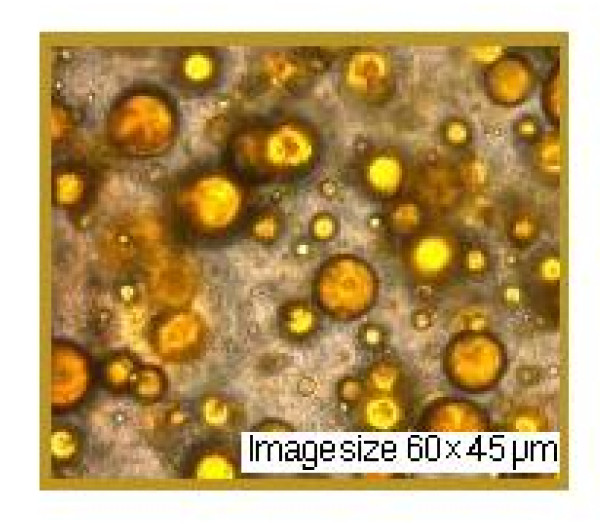
**Nanoemulsion 2 layer (aqueous extract of propolis and lycopene) after two week**.

**Figure 8:**
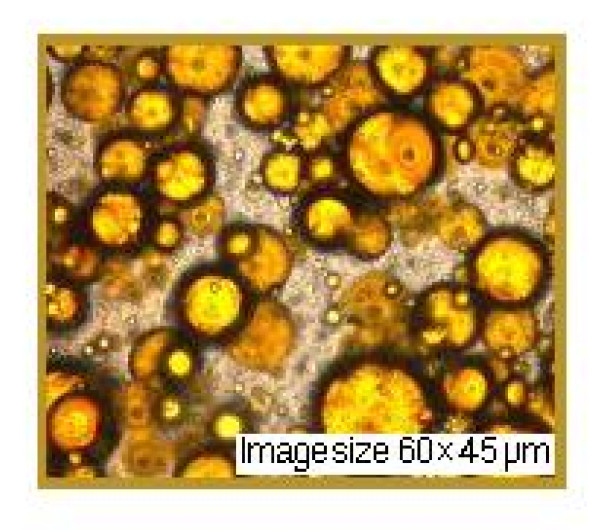
**Nanoemulsion 2 layer (aqueous extract of propolis and lycopene) after three weeks**.

This experiment was conducted to determine the photoprotection capacity of natural substances in nanoemulsions. In both nanoemulsions we also introduced a UVR-absorber (Saliform), accepted by European standards, to observe its influence on the analyzed samples. In Table 3 presents the absorbance's of two nanoemulsions measured by UVA, UVA/UVB ratio and SPF. The obtained data show that most absorbance in the UV domain in the relative parameter 5 is present in nanoemulsion 2+ absorber-UVR (Saliform) 1:1 (v/v). Regarding UVA/UVB ratio with the relative parameter 0.82, most absorbance is found in the same nanoemulsion and in the case of SPF with absolute parameter 10.9, in nanoemulsion 2. As shown by these results, highest absorbance parameters may be assigned to nanoemulsion 2. Experimental formulations showed a pseudo-plastic rheology, under the influence of shear stress (Figure 5). The viscosity is directly dependent on the formulations content of propolis. Microscopic observations of the experimental nanoemulsions confirmed formation of spherical particles, as shown in Figure 5. Differences in droplet size of these formulations were not statistically significant (droplet size was found to be 45 μm). Nanoemulsions were kept for three weeks at 4°C in order to provide a stable formulation for local application. Nanoemulsion stability is shown in Figure 6, 7 and 8. This study suggests that the nanoemulsions made of aqueous extract of propolis and lycopene and prepared with active substances may confer better therapeutic effects than conventional formulations, as a result of dose controlled local release longer period of time, which could lead to a greater efficiency and to a greater acceptance by the skin (i.e. better compliance). These results are supported by presented data in Table 4. Collagenase (inactive form of pre-collagenase, activated by trypsin) is an enzyme that cleaves the peptide bonds of fibrillar collagen types I and III. Experimental results showed that both nanoemulsions induced a reduction in collagenase activity. The intensity of anti-inflammatory effect varies with time interval covered by the assessment of therapeutic response according to data in Table 4. The activity of propolis and lycopene nanoemulsions (Table 5) was emphasized by measuring the induced inflammation. Produced inflammation decreased by 75% for nanoemulsion 1 and 100% for nanoemulsion 2. The maximal inflammation reduction effect occurred after 8 hours from the initial application. Preliminary tissue examination showed that these formulations did not produce irritation. Local application of the experimental nanoemulsion (propolis and lycopene) has great potential, both in terms of efficacy (analgesia) and therapeutic safety. Nanoemulsions have proved a better therapeutic efficacy compared to standard suspension, was observed improving monitored parameters for a longer period of time (24 hours). These systems can be seen as a viable alternative to conventional creams, due to their ability to improve residence time and thereby, bioavailability.

**Table 3:** UVA radiation, the UVA/UVB ratio and nanoemulsion SPF with and without UVR-absorber

No.	Samples	UVA	UVA/UVB	SPF
1	Nanoemulsion 1+absorber-UVR (Saliform)1:1 (v/v)	4.0	0.71	5.9

2	Nanoemulsion 2 +absorber-UVR (Saliform)1:1 (v/v)	5	0.82	6.9

3	Nanoemulsion 1	3.1	0.24	9.1

4	Nanoemulsion 2	3.2	0.815	10.9

**Table 4:** Enzymatic activity of collagenase in the presence of nanoemulsions

Substance	Concentration (μg/ml)	Enzymatic activity (Units/mg of protein)	% Inhibition
Nanoemulsion 1	40	1.205 ± 0.001	26.81

Nanoemulsion 2	40	1.033 ± 0.003	37.14

Without nanoemulsion	-	1.648 ± 0.007	-

Reaction conditions: T = 25°C, pH 7.5, λ = 345 nm, t = 5 min.			

**Table 5:** Activity of aqueous extract of propolis and lycopene assessed on induced mouse paw edema

Compound	Mean ± SE difference in right and left paw volumes (ml)				Reduction of edema (%)			
	
	4h	6h	8h	24h	4h	6h	8h	24h
Control	0.147 ± 0.02	0.186 ± 0.11	0.246 ± 0.03	0.165 ± 0.01	-	-	-	-

Regular sunscreen	0.039 ± 0.03	0.026 ± 0.10	0.020 ± 0.14	0.015 ± 0.004	69	67	72	73

Nanoemulsion 1	0.019 ± 0.03	0.014 ± 0.01	0.011 ± 0.02	0.007 ± 0.005	97	85	78	75

Nanoemulsion 2	0.013 ± 0.04	0.008 ± 0.02	0.003 ± 0.02	0.001 ± 0.00	99	99	99	100

## Discussion

Emulsions are a mixture of molecules in a combination of two liquids that keep their properties unaltered. This feature has been used in the delivery of poorly soluble drugs [26]. Nanoemulsions have a greater capacity for micellar solubilisation compared to simple solutions and offer advantages in thermodynamic stability to unstable dispersions (suspensions), as can be produced with less energy input and have a greater shelf life [27]. The nanoemulsions are systems with droplet sizes of approximately 45 μm, having surfactant ratios of 47/53 and respectively 70/30 of aqueous extract of propolis-lycopene. UVA absorbance with relative parameter 5 is manifested by nanoemulsion 2 +absorber-UVR (Saliform) 1:1 (v/v), in the case of the UVA/UVB ratio with relative parameter 0.82 by the same nanoemulsion and in the case of SPF with absolute parameter 10.9 by the nanoemulsion 2. Highest absorbance parameters can be assigned to nanoemulsion 2. Prepared formulations showed a pseudo-plastic rheology, under the influence of shear stress. It appears that viscosity is directly dependent on the propolis content of the formulation. Lycopene is insoluble in water; it can be dissolved only in organic solvents and oils [28-30]. Researchers have correlated the antioxidant function of lycopene (ability to protect cells and other body structures caused by oxidative damage) with the protection of DNA (our genetic material) inside the white blood cells [31].

White blood cells (WBC) are mediators of inflammation and the immune response. Unlike other food phytonutrients, whose effects have only been studied in animals, lycopene from tomatoes has been repeatedly studied in humans, where research has shown additional protection against many types of diseases [32,33].

## Conclusions

The experimental nanoemulsions in a high kinetic stability, and reduction in collagenase activity by 37.14% for a 70/30 surfactant ratio and respectively 26.81% for a 47/53 ratio. These nanoemulsions provided a sustained drug release *in vitro *for a period of 8 hours. Lycopene antioxidant as a nanoemulsion component beside its moisturizer characteristic improves the ability of the skin to defend against sunlight.

## Methods

### Aqueous extract of propolis

Aqueous extract of propolis was obtained by refluxing 100 g of propolis powder and 250 ml of double distilled water. It was concentrated in a water bath then filtered resulting in 1.5 cm^3 ^of extract with 95% of dry substance.

### Determination of lycopene

Lycopene was obtained from ripe tomatoes (*Lycopersicon esculentum*) by solvent extraction. Samples were homogenized in a laboratory homogenizer. 5ml 0.05% BHT in acetone, 5 ml of ethanol and 10 ml of hexane were added to 0.6 g homogenated sample. The supplemented homogenate was kept on ice and stirred with a magnetic stirrer for 15 minutes. Then 3 ml of deionized water were added and samples were mixed for additional 5 minutes. Samples were then left at room temperature for 5 minutes to allow phase separation. The absorption of the hexane layer (upper layer) was measured in a 1cm quartz cuvette at a wavelength of 503 nm, against hexane as blank. Lycopene was measured quantitatively by UV-VIS spectrophotometer T60U, PG Instruments Limited, UV WIN^®^version 5.05; detection was performed at 503 nm and calculated using the following formula:

Absorbance at 503 nm (A_503_) =

ε (M^-1^·cm^-1^)·b(cm)

[Lycopene concentration (M)]

The measuring conditions were: scan speed 90 nm/min and an interval of 1 nm. After extraction, was hexane evaporated to dryness in a vacuum evaporator, under a nitrogen stream [34]. All substances were purchased from Sigma Chemical.

### Preparation of nanoemulsions

Nanoemulsions were prepared by adding lycopene to aqueous solution of propolis (50 mg propolis in 10 ml D.W.), using a magnetic stirrer at ~ 2000 rpm. The mixture was introduced for in an ultrasonic bath at 20 kHz 20 minutes. The nanoparticles that are formed have a lycopene-propolis loaded shape. After ultrasonic treatment the solution is brought at room temperature (22°C).

The excess organic solvent in excess was evaporated using a rotary evaporator and samples were kept for further analysis by lyophilisation. Independent of preparation temperature, samples were kept at 25°C [35].

### Fourier transforms infrared spectrometry (FTIR)

For spectral characterization (FTIR spectrometry), samples were prepared as follows: compounds obtained after heat treatment were mixed at a temperature of 1300°C with potassium bromide powder, previously dried for 24 hours at a temperature of 120°C, at a mass ratio of 0.04:1. After a vigorous mix to obtain uniformisation, pills with a thickness of 0.5-0.75 mm and 13 mm in diameter at a pressure of 0.3 GPa in normal atmosphere were prepared. The pills were analyzed using the *JASCO 660 PLUS *spectrophotometer, which recorded the IR absorption spectra in the area 4000 cm^-1 ^100-400 cm^-1^. For *in vitro *characterization of the experimental nanoemulsions, permeability studies and rheological measurements were carried out [36].

### Permeability studies

Membrane permeability of the experimental nanoemulsions was investigated by filling the donor compartment of a diffusion cell with a 2 g test mixture. All other experimental conditions identical to those described for permeability studies of solid systems [37].

### Viscosity measurement

Apparent viscosity of the experimental nanoemulsions was determined using a viscometer Brookfield Rheostress DV-III + Rheometer. Measurements were performed 3 times at 25°C using SC4 spindle. To determine the influence of shear stress applied to the microstructure of the prepared nanoemulsions, measurements were made at a rotation speed of 1 and 10 rpm.

Apparent viscosity of the controlled product (2% HPMC dispersion in water), were examined under similar conditions [38].

### Enzyme activity measurements

Enzyme activity is determined by a continuous spectrophotometric method, using as substrate 2-L-leucylglycyl furanacryloyl-L-prolyl-L-alanine (FALGPA, a specific collagenase substrate), as it is preferentially hydrolyzed much faster than other synthetic substrates.

Measurement of the substrate absorbance decrease was done at 345 nm.

### Pharmacological evaluation of formulations

Identification and quantification are done according to the methods described in the European Directorate for the Quality of Medicines [39].

### Animal testing

Preparation: adult, young, healthy animals of the species of guinea pigs, breed albino were used. The animals were acclimatized to laboratory conditions for at least five days before test. Animals were divided randomly into treatment and control groups before test. Their skin was cleaned by clipping, shaving or, if possible, by chemical depilation without excoriation (cleaning method is based on the test method used). Animals have been weighed before and after test.

#### Experimental procedure

Superficial skin burns were induced using a UV radiation lamp.

#### Testing the anti-inflammatory action

Assessment of anti-inflammatory action was achieved by evaluating the inhibition of rat paw edema induced by a 2% solution of carrageenan.

The percentage inhibition of edema was calculated using the following equation:

Inhibition (%) =

$$\frac{\text{Mean paw diameter (control) - Mean paw diameter (treated) }}{\text{Mean paw diameter (control)}}\cdot 100$$

The edema inhibition rate of each group was calculated as follows:

Inhibition (%) =

$$\frac{\text{Mean number of writhing (control)-Mean of writhing (test) }}{\text{Mean number of writhing (control)}}\cdot 100$$

Monitoring and staging: approximately 21 hours after the patch removal, hair is cleared off the surface exposed to challenge concentration. After 3 hours (about 30 hours after the challenge patch application) skin reactions were observed and recorded. After an additional time of 24 hours (54 hours) skin reactions were observed and recorded again. "Blind" reading is recommended for tested and control animals. All skin reactions and any unusual results, including systemic reactions caused by induction and challenge procedures were observed and recorded in accordance with the Magnusson/Kligman staging. If any of the reactions are difficult to interpret, other procedures can be taken into account, for instance histopathological examination or measurements of the skin fold.

Staging Magnusson/Kligman scale for assessing the post-challenge responses: 0 = no visible change, 1 = erythema or discrete form of spot, 2 = moderate and confluent erythema, 3 = intense erythema and swelling [40]

### Determination of in vitro sun protection factor (SPF)

Determination of SPF (sun protection factor) was performed using a spectrophotometer, equipped with an integrating sphere, with appropriate software and a TRANSPOR 3 TM support, with a composition similar to that of natural skin, on which the amount of 2 mg/cm^2 ^of nanoemulsion was applied [40].

### Statistical analysis

Values were expressed as mean ± S.D. Statistical significance was evaluated by Students-„t‟ test at 5% level of significance (p < 0.05).

## Competing interests

The authors declare that they have no competing interests. The opinions expressed in this article are those of the authors and do not necessarily represent any agency determination or policy.

## Authors' contributions

CG participated in the design of the study and performed the statistical analysis. MB conceived of the study, and participated in its design and coordination. All authors read and approved the final manuscript.
